# Subtype prediction of intrahepatic cholangiocarcinoma using dynamic contrast-enhanced ultrasound

**DOI:** 10.1186/s13244-024-01683-y

**Published:** 2024-05-16

**Authors:** Ming-Rui Zhu, Chong-Ke Zhao, Yi-Kang Sun, Xiao-Long Li, Hao-Hao Yin, Dan Lu, Xin Ye, Xin-Yuan Hu, Xi Wang, Han-Sheng Xia, Hong Han, Bo-Yang Zhou, Hui-Xiong Xu, Li-Fan Wang

**Affiliations:** 1 Department of Ultrasound, Institute of Ultrasound in Medicine and Engineering, Zhongshan Hospital, Fudan University, 200032 Shanghai, China; 2 Shanghai Institute of Medical Imaging, 200032 Shanghai, China; 3 School of Medicine, Anhui University of Science and Technology, 232000 Anhui, China

**Keywords:** Intrahepatic cholangiocarcinoma, Dynamic contrast-enhanced ultrasound, Subtype, Predictive model

## Abstract

**Objective:**

The study aimed to investigate the predictive value of dynamic contrast-enhanced ultrasound (DCE-US) in differentiating small-duct (SD) and large-duct (LD) types of intrahepatic cholangiocarcinoma (ICC).

**Methods:**

This study retrospectively enrolled 110 patients with pathologically confirmed ICC lesions who were subject to preoperative contrast-enhanced ultrasound (CEUS) examinations between January 2022 and February 2023. Patients were further classified according to the subtype: SD-type and LD-type, and an optimal predictive model was established and validated using the above pilot cohort. The test cohort, consisting of 48 patients prospectively enrolled from March 2023 to September 2023, was evaluated.

**Results:**

In the pilot cohort, compared with SD-type ICCs, more LD-type ICCs showed elevated carcinoembryonic antigen (*p* < 0.001), carbohydrate antigen 19-9 (*p* = 0.004), ill-defined margin (*p* = 0.018), intrahepatic bile duct dilation (*p* < 0.001). Among DCE-US quantitative parameters, the wash-out area under the curve (WoAUC), wash-in and wash-out area under the curve (WiWoAUC), and fall time (FT) at the margin of lesions were higher in the SD-type group (all *p* < 0.05). Meanwhile, the mean transit time (mTT) and wash-out rate (WoR) at the margin of the lesion were higher in the LD-type group (*p* = 0.041 and 0.007, respectively). Logistic regression analysis showed that intrahepatic bile duct dilation, mTT, and WoR were significant predictive factors for predicting ICC subtypes, and the AUC of the predictive model achieved 0.833 in the test cohort.

**Conclusions:**

Preoperative DCE-US has the potential to become a novel complementary method for predicting the pathological subtype of ICC.

**Critical relevance statement:**

DCE-US has the potential to assess the subtypes of ICC lesions quantitatively and preoperatively, which allows for more accurate and objective differential diagnoses, and more appropriate treatments and follow-up or additional examination strategies for the two subtypes.

**Key Points:**

- Preoperative determination of intrahepatic cholangiocarcinoma (ICC) subtype aids in surgical decision-making.
- Quantitative parameters from dynamic contrast-enhanced US (DCE-US) allow for the prediction of the ICC subtype.
- DCE-US-based imaging has the potential to become a novel complementary method for predicting ICC subtypes.

**Graphical Abstract:**

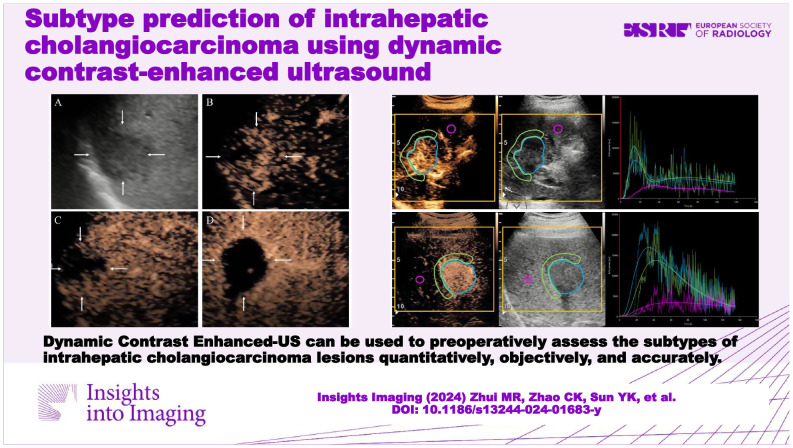

## Introduction

Intrahepatic cholangiocarcinoma (ICC), the second most common primary liver malignancy (about 15%) following hepatocellular carcinoma (HCC), has been increasingly detected in patients in recent years [1]. Owing to the high aggressiveness, the 5-year overall survival rates of patients with ICC are generally no more than 30%–50%, and the postoperative recurrence rate is as high as 60%–70% [2].

ICC is a heterogeneous group of malignancies originating from the epithelial cells of the intrahepatic bile duct, which can be classified into two subtypes in terms of different origin levels, small-duct (SD) type, and large-duct (LD) type [3]. Most of the LD-type ICCs are anatomically located around the second branches of portal veins in the peripheral liver parenchyma, whose growth patterns are relatively diverse, with more periductal-infiltrating type and intraductal-growing type [4]. However, most SD-type ICCs are located in the peripheral liver parenchyma and most of them are mass-forming type [5]. Meanwhile, 91% of LD-type ICCs had at least focal periductal infiltration. The more invasive biological behavior of LD-type ICC leads to a worse prognosis and lower 5-year overall survival rates than that of SD-type ICC [1, 6]. Due to the fact that radical surgical resection is currently the only possible treatment to care for ICC and the 5-year postoperative recurrence rate is high, accurate identification of ICC subtypes may have greater significance for management, such as choosing more optimal treatments, better evaluating the difficulty of surgery, and making follow-up strategies. In view of the relatively higher infiltrative nature, preoperative subtype prediction can provide a basis for expanding the surgical resection range for patients with LD-type ICC, which may make the surgery more thorough. Meanwhile, due to the stronger invasiveness of LD-type ICC, it is necessary to conduct different follow-up or additional examination strategies for the two subtypes. For example, the interval between follow-up for LD-type could be appropriately shortened, such as every 3 months within 2 years after resection, and every 3–6 months for 2 to 5 years after resection [7]. Based on this, patients with different subtypes can receive more accurate and comprehensive treatment strategies, which is also a shift to personalized treatment and precision medicine. Although biopsy can acquire pathological diagnosis for ICC subtypes preoperatively, it still has the disadvantage of the potential risk of needle tract seeding [8]. Therefore, an effective and non-invasive tool is expected to predict ICC subtypes preoperatively.

In recent years, several studies have shown that the subtype of ICCs can be predicted by analyzing the CT or MRI features. On MRI, Park et al reported that infiltrative contour, diffuse biliary dilatation, no arterial phase hyperenhancement (APHE), and vascular invasion suggested the LD-type and Rhee et al reported that the presence of biliary diffuse dilatation and abnormality were significant features suggestive of the LD-type [9, 10]. Recently, Xiao et al also reported that arterial phase hypoenhancement, tumor in the vein, intrahepatic duct dilatation, lack of targetoid appearance in T_2_WI, and lack of targetoid restriction were predictors of LD-type ICCs [11]. On CT, Fujita et al reported that LD-type was more hypovascular, invasive, and rim-APHE, while Nam et al reported that APHE, round or lobulated contour, and lack of bile duct encasement were associated with the SD-type [12, 13].

Contrast-enhanced ultrasound (CEUS) is an improvement to conventional ultrasound (US) for the characterization of focal hepatic lesions, which can be used to differentiate intrahepatic nodules detected by gray-scale US but not definitely diagnosed [14, 15]. The contrast agent of CEUS is usually a pure blood pool contrast agent, and CEUS can continuously monitor the blood perfusion in real time [16]. Therefore, CEUS plays a good complementary role in the timely diagnosis of diseases. However, owing to its disadvantages such as relying on the experience of radiologists, the outcome of CEUS is relatively subjective, which results in inter-observer variability [17]. Therefore, a more objective and stable evaluation method of CEUS is needed. CEUS quantitative analysis software such as VueBox® can quantitatively analyze the data based on CEUS cines, which provides intuitive, quantitative, and visual parameters for the diagnosis, differential diagnosis between benign and malignant lesions, and follow-up after treatment of many lesions [18–24]. We hypothesized that dynamic contrast-enhanced ultrasound (DCE-US) has potential clinical value in the differentiation of the ICC subtypes.

## Materials and methods

### Patients

The study was approved by the Ethics Committee of the institution (No: B2022-569R) and informed consent was waived.

**Fig. 1:**
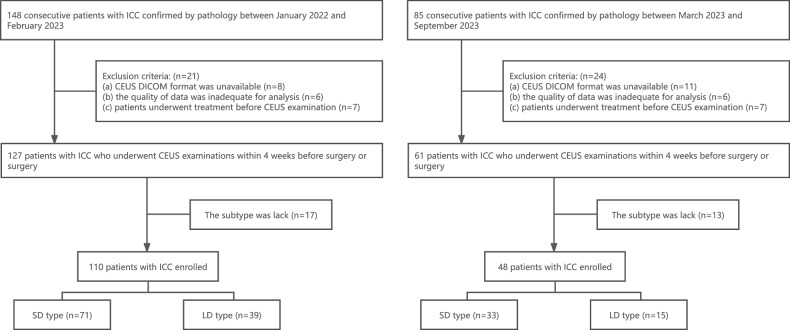
Patient selection flowchart. Left is the pilot cohort and right is the test cohort. ICC, intrahepatic cholangiocarcinoma; EMR, electronic medical record; CEUS, contrast-enhanced ultrasound; SD, small-duct type of ICC; LD, large-duct type of ICC

Between January 2022 and February 2023, 148 patients with ICCs confirmed by pathology were retrospectively enrolled as the pilot cohort. From March 2023 to September 2023, 85 patients were prospectively enrolled in the test cohort. The inclusion criteria were: (a) patients with ICC lesions confirmed by pathology; (b) patients who underwent preoperative CEUS examinations within 4 weeks before surgery or biopsy. The exclusion criteria were as follows: (a) the DICOM format of CEUS was unavailable; (b) the quality of CEUS data was inadequate for analysis; (c) patients underwent any treatment before CEUS examination; (d) the definite pathological subtype of ICC was lack. The largest lesion was chosen as the target for analysis if a patient had multiple lesions. Finally, a total of 110 and 48 patients were enrolled in the pilot cohort (71 SD-type group and 39 LD-type group) and the test cohort (33 SD-type group and 15 LD-type group), respectively. Figure 1 shows the patient selection flowchart.

### US and CEUS data acquisition

All patients were subject to both conventional US and CEUS examinations by experienced US radiologists using Samsung RS80A (Samsung Ultrasound System, Seoul, Korea) with a C1-6 convex transducer, Mindray Resona 7 s (Mindray Medical System, Shenzhen, China) with a SC5-1U convex transducer, and EPIQ7 (Philips Healthcare, Bothell, WA, USA) with a 5-1 MHz convex transducer. The whole liver was scanned by the conventional US first, and then the location, size, echogenicity, and margin of lesions were observed. A low mechanic index (MI, 0.08–0.12) pattern of CEUS was performed to observe the targeted lesion. A volume of 2.0 mL contrast agent (SonoVue, Bracco SpA, Milan, Italy) was injected in the bolus via the antecubital vein and followed by a 5-mL saline flush. After contrast medium injection, the enhancement features of suspected ICC lesions were recorded during the arterial phase (AP), portal venous (PVP), and late phases (LP) based on guidelines. A clip of at least 2 min was recorded continually without moving the transducer, and then scanned at 20–30 s intervals and recorded for 5 min or until the contrast agent disappeared. All imaging data in DICOM format were stored for further analysis.

### US and CEUS analysis

All conventional US and CEUS were independently reviewed by two experienced abdominal US radiologists (with more than 10 years of experience in the abdominal CEUS), who were blinded to the pathological diagnosis and medical history. Any discrepancies between the two US radiologists were solved with a consensus by discussion.

These conventional US features were assessed: (a) location (peripheral, or perihilar); (b) lesion size; (c) echogenicity (hyper-, iso-, hypo-, or mixed-echogenicity, compared with surrounding liver parenchyma); (d) lesion margin (well-, or ill-defined); (e) hepatic background (normal, fatty liver or cirrhosis); (f) intrahepatic bile duct dilation (presence or absence), and (g) hepatolith (presence or absence). On CEUS, the arterial phase (AP) (0–30 s after the injection), portal vein phase (PVP) (31–120 s after the injection), and late phase (LP) (121–240 s after the injection) are defined. Then, the following CEUS features were evaluated: (a) AP enhancement pattern (APHE, rim-APHE, no APHE); (b) time of enhancement onset, peak, and wash-out onset; (c) degree of wash-out (no, mild, or marked) in PVP and LP; (d) enhancement degree of PVP and LP (hyperenhancement, isoenhancement, or hypoenhancement, compared with enhancement degree of surrounding liver parenchyma).

### DCE-US analysis

After obtaining the DICOM format of CEUS, the cines were transferred to another offline computer and were analyzed using VueBox® software (Bracco Imaging, Milan, Italy) by another US radiologist (more than 8 years of experience in the abdominal CEUS) who was also blinded to the pathological diagnosis and clinical history. ICC lesions were observed dynamically, and three regions of interest (ROIs) were placed manually: around the whole ICC lesion, at the margin of the lesion, and in the surrounding liver parenchyma accordingly. The depth from the body surface to the three ROIs was kept the same, and attention was paid to avoid the surrounding major blood vessels and necrosis in the tumor. Meanwhile, the motion compensation function was used to reduce the breath motion artifact.

A time-intensity curve (TIC) was then generated by VueBox®, describing the dynamic process of wash-in and wash-out of the microbubbles in the ROIs, which should be observed in shape, peak intensity, and area under the curve (AUC). Then, relevant quantitative parameters of CEUS were obtained through curve fitting, whose results were considered credible when the quality of fit (QOF) > 75%. These extracted quantitative parameters included: mean contrast signal intensity (MeanLin), peak enhancement (PE), rise time (RT), time to peak (TTP), mean transit time (mTT), fall time (FT), wash-in rate (WiR), wash-out rate (WoR), wash-in area under the curve (WiAUC), wash-out area under the curve (WoAUC), wash-in and wash-out area under the curve (WiWoAUC) and wash-in perfusion index (WiPI).

### Statistical analysis

Normal distribution was evaluated using the Kolmogorov–Smirnov test. The Chi-square test or Fisher exact test was used for categorical variables, which were presented as numbers and percentages. Continuous variables, expressed as the mean ± standard deviation when conforming to normal distribution, and median (interquartile range) when not, Student *t*-test or Mann–Whitney *U*-test was used when it is appropriate. The consistency of quantitative parameters between different equipment was tested using the independent-samples Kruskal–Wallis test (Supplement Table 1). In the pilot cohort, potential predictive parameters were identified through binomial logistic regression analysis for the differentiation of ICC subtypes, and a predictive model was developed based on three significant factors. The predictive model was validated in the pilot cohort, and then the diagnostic performance of the predictive model in the test cohort was also evaluated using receiver operating characteristic (ROC) curve analysis. The cut-off value was calculated using the Youden index, and then the sensitivity, specificity, positive predictive value (PPV), negative predictive value (NPV), and accuracy were calculated. Statistical significance was set at a *p*-value of less than 0.05. Statistical analyses were performed using the software programs IBM SPSS Statistics 27.0 (IBM SPSS Inc., Chicago, USA) and Origin 2022 (OriginLab, Massachusetts, USA).

## Results

### Demographics and clinical characteristics

A total of 110 patients (mean age 61.2 ± 10.2 years, range 23–82 years) with 110 ICC lesions were enrolled in the pilot cohort in this study, in which 71 lesions (64.5%) were classified as SD-type and 39 lesions (35.5%) as LD-type. For the test cohort, a total of 48 patients (mean age 62.3 ± 10.0 years, range 42–78 years) including 33 SD-type (68.7%) and 15 LD-type (31.3%) were prospectively enrolled. There was no significant difference in sex, age, and history of hepatitis between the two subtypes (*p* > 0.05), both in the pilot cohort and test cohort. The size of the nodule showed a significant difference in the test cohort (*p* = 0.016) but showed no difference in the pilot cohort (*p* = 0.771). There were 14 (35.9%) LD-type patients and 7 (9.9%) SD-type patients with elevated carcinoembryonic antigen (CEA) (> 5 ng/mL, *p* < 0.001). The carbohydrate antigen 19-9 (CA19-9) (> 34 U/mL) was elevated in 26 (66.7%) LD-type patients and 27 (38.0%) SD-type patients (*p* = 0.004). Similar findings were obtained in the test cohort, including CEA (*p* = 0.006) and CA19-9 (*p* = 0.034). The details are shown in Table 1.

**Table 1:** Comparison of clinical characteristics

Variable	Pilot cohort (*n* = 110)			Test cohort (*n* = 48)		
	SD (*n* = 71)	LD (*n* = 39)	*p*_1_-value	SD (*n* = 33)	LD (*n* = 15)	*p*_2_-value
Age, years^a^	60.08 ± 10.63	63.15 ± 9.14	0.239	61.58 ± 10.71	63.80 ± 9.89	0.480
Male/female	46/25	19/20	0.101	22/11	7/8	0.194
Nodule size (IQR), mm^b^	42 (28, 59)	45 (32, 52)	0.771	48 (28, 60)	67 (47, 90)	0.016*
History of hepatitis	28 (39.4%)	11 (28.2%)	0.236	8 (24.2%)	2 (13.3%)	0.393
Preoperative tumor maker						
AFP > 20, ng/mL	7 (9.9%)	3 (7.7%)	0.705	3 (9.1%)	1 (6.7%)	0.872
CEA > 5, ng/mL	7 (9.9%)	14 (35.9%)	< 0.001*	2 (6.1%)	6 (40.0%)	0.006*
CA19-9 > 34, U/mL	27 (38.0%)	26 (66.7%)	0.004*	4 (12.1%)	12 (80.0%)	0.034*

Data in parentheses are percentages except for special indications

*SD* small-duct type of ICC, *LD* large-duct type of ICC, *AFP* alpha-fetoprotein, *CEA* carcinoembryonic antigen, *CA19-9* carbohydrate antigen 19-9

* *p*_1_-value has a significant difference between SD and LD-type ICCs in the pilot cohort, or *p*_2_-value has a significant difference between SD and LD-type ICCs in the test cohort

^a^ Data are presented as the mean ± standard deviation

^b^ Data are presented as median (interquartile range)

### Conventional US and CEUS features

**Fig. 2:**
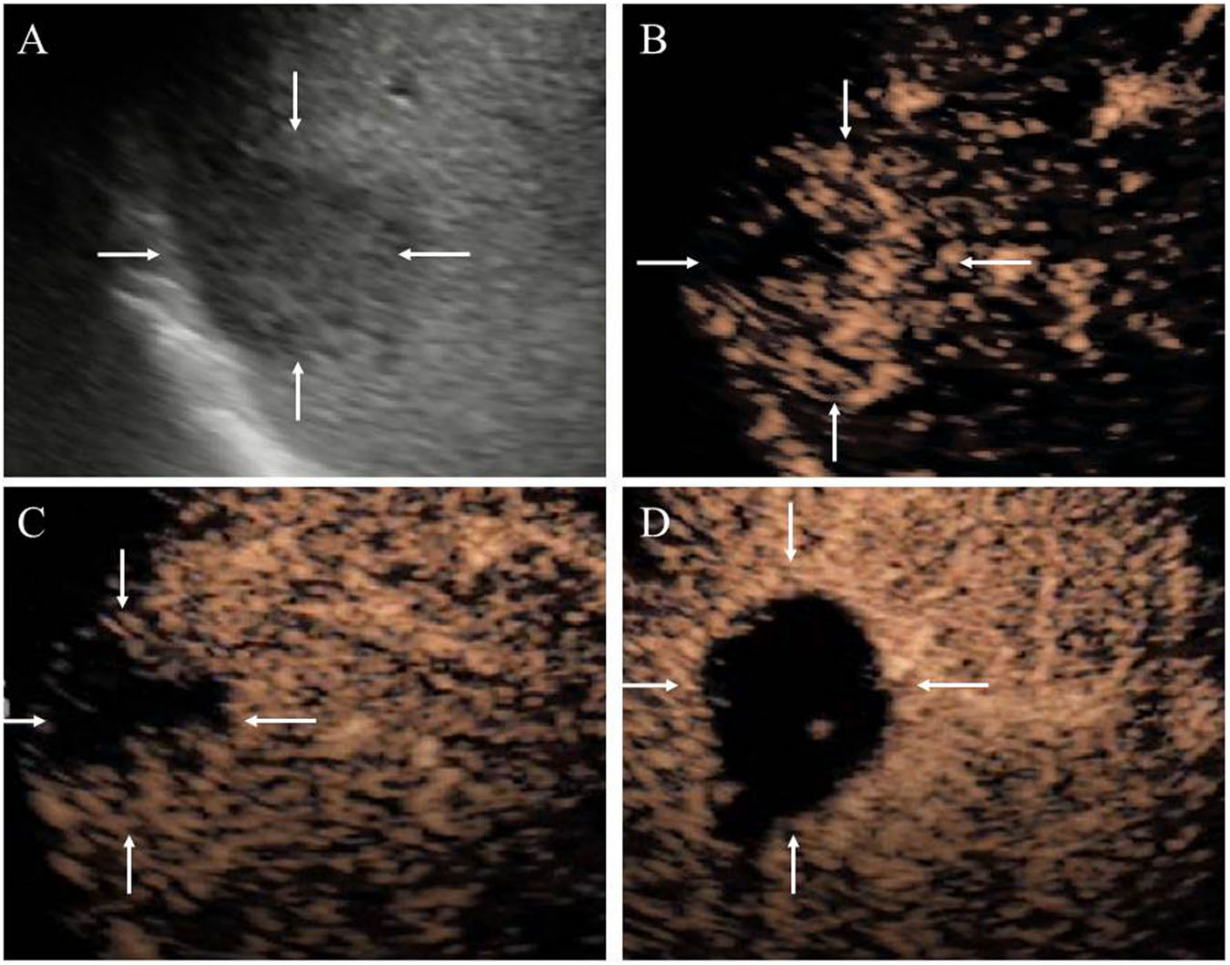
A 59-year-old woman with a 33 mm intrahepatic cholangiocarcinoma (ICC) lesion which is confirmed by pathology as SD-type. **A** B-mode ultrasound shows a hypo-echoic lesion (white arrow) with an ill-defined boundary located in the peripheral area. **B** Arterial phase rim hyperenhancement (rim-APHE, white arrow) is observed at 22 s after contrast agent injection. **C** Early wash-out is observed at 38 s (white arrow). **D** Marked wash-out is present at 65 s (white arrow)

**Fig. 3:**
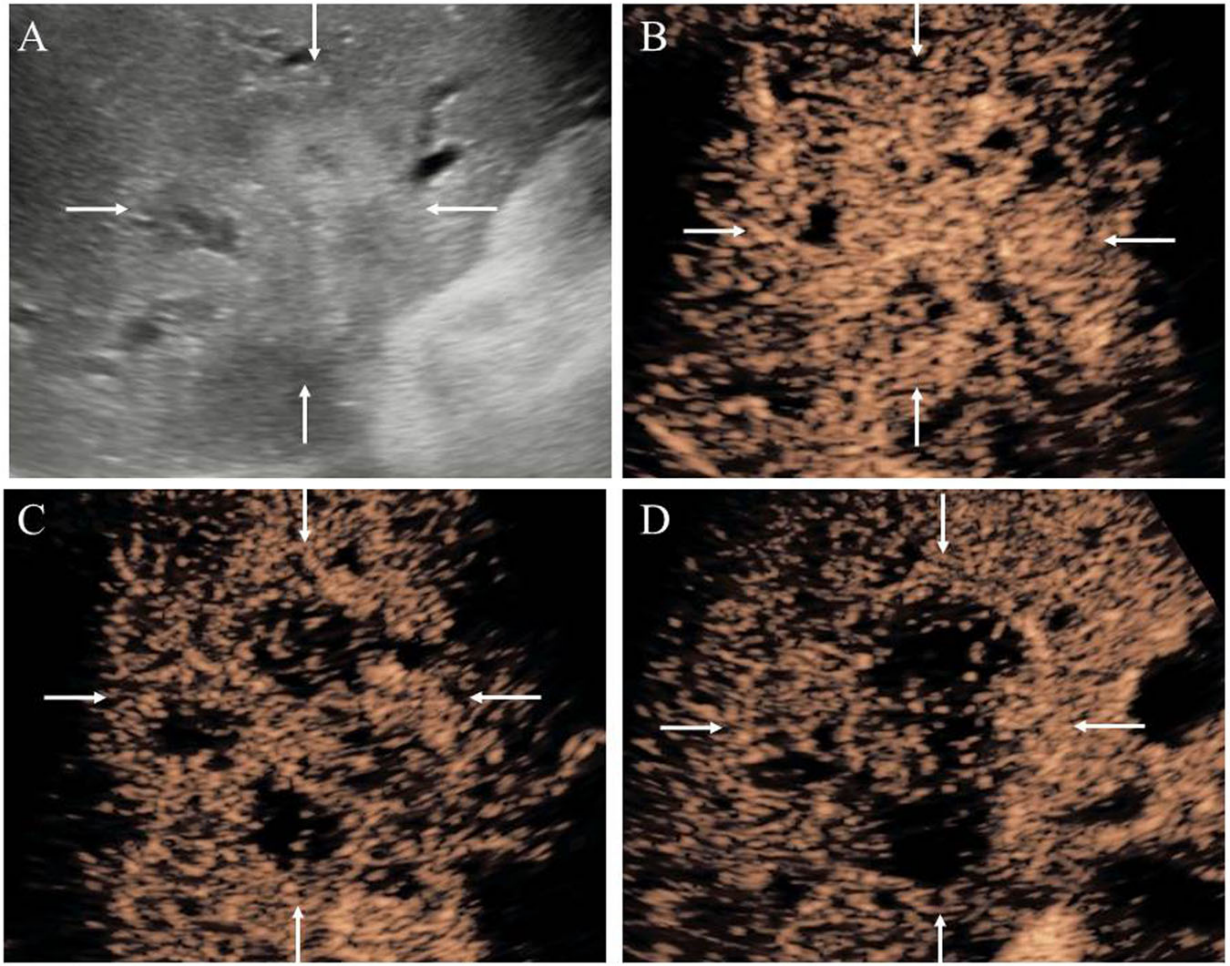
A 50-year-old woman with a 35 mm intrahepatic cholangiocarcinoma (ICC) lesion which is confirmed by pathology as LD-type. **A** B-mode ultrasound shows a hyper-echoic lesion (white arrow) with ill-defined boundary located in the perihilar area. **B** Arterial phase hyperenhancement (APHE, white arrow) is observed at 17 s after contrast agent injection. **C** Early wash-out is observed at 29 s (white arrow). **D** Marked wash-out is present at 85 s (white arrow)

By analysis of conventional US and CEUS features for the prediction of LD-type over SD-type ICCs, significant differences were noted in the ill-defined margin of the lesion (97.4%, 38/39 vs. 78.9%, 56/71, *p* = 0.018) and the presence of intrahepatic bile duct dilation (56.4%, 22/39 vs. 11.3%, 8/71, *p* < 0.001) in the pilot cohort. There were no significant differences in location, echogenicity, hepatic background, the presence of hepatolith, AP enhancement pattern, enhancement onset time, AP peak time, wash-out onset time, wash-out degree, PVP enhancement degree, and LP enhancement degree (all *p* > 0.05) between SD-type and LD-type ICCs. In the test cohort, the presence of intrahepatic bile duct dilation (53.5%, 8/15 vs. 15.2%, 5/33, *p* = 0.006) also differed significantly between the two subtypes (Table 2). The conventional US and CEUS imaging of SD-type and LD-type ICCs are shown in Figs. 2 and 3.

**Table 2:** The univariate analysis of conventional US features and CEUS features

Variable	Pilot cohort			Test cohort		
	SD (*n* = 71)	LD (*n* = 39)	*p*_1_-value	SD (*n* = 33)	LD (*n* = 15)	*p*_2_-value
Location			0.076			0.295
Peripheral	55 (77.5%)	24 (61.5%)		30 (90.9%)	12 (80.0%)	
Perihilar	16 (22.5%)	15 (38.5%)		3 (9.1%)	3 (20.0%)	
Echo intensity			0.416			0.188
Hypo-	57 (80.3%)	26 (66.7%)		25 (75.8%)	9 (60.0%)	
Iso-	2 (2.8%)	1 (2.6%)		0 (0.0%)	2 (13.3%)	
Hyper-	10 (14.1%)	10 (25.6%)		6 (18.2%)	3 (20.0%)	
Mixed-	2 (2.8%)	2 (5.1%)		2 (6.1%)	1 (6.7%)	
Margin			0.018*			0.037*
Well-defined	15 (21.1%)	1 (2.6%)		8 (24.2%)	0 (0.0%)	
Ill-defined	56 (78.9%)	38 (97.4%)		25 (75.8%)	15 (100.0%)	
Hepatic background			0.463			0.538
Normal	29 (40.8%)	16 (41.0%)		15 (45.5%)	7 (46.7%)	
Fatty liver	17 (23.9%)	13 (33.4%)		11 (33.3%)	3 (20.0%)	
Cirrhosis	25 (35.2%)	10 (25.6%)		7 (21.2%)	5 (33.3%)	
The presence of intrahepatic bile duct dilation	8 (11.3%)	22 (56.4%)	< 0.001*	5 (15.2%)	8 (53.3%)	0.006*
The presence of hepatolith	0 (0.0%)	0 (0.0%)	1.000	0 (0.0%)	0 (0.0%)	1.000
AP Enhancement pattern			0.729			0.669
Rim APHE	25 (35.2%)	14 (35.9%)		9 (27.3%)	5 (33.3%)	
APHE	43 (60.6%)	22 (56.4%)		24 (72.7%)	10 (30.3%)	
No APHE	3 (4.2%)	3 (7.7%)		0 (0.0%)	0 (0.0%)	
Enhancement onset time, s^a^	17.45 ± 5.16	18.26 ± 3.21	0.095	20.91 ± 4.93	17.87 ± 4.60	0.078
AP peak time, s^a^	26.76 ± 5.57	28.03 ± 5.06	0.117	28.55 ± 5.90	27.07 ± 6.89	0.422
Wash-out onset time (IQR), s^b^	40 (37, 47)	44 (38,50)	0.308	40 (36, 45)	41 (32,55)	0.920
Wash-out degree			0.273			0.636
No	1 (1.4%)	1 (2.6%)		0 (0.0%)	0 (0.0%)	
Mild	26 (36.6%)	20 (51.3%)		13 (39.4%)	7 (46.7%)	
Marked	44 (62.0%)	18 (46.2%)		20 (60.6%)	8 (53.3%)	
PVP enhancement degree			1.000			1.000
Hypo-	71 (100%)	39 (100%)		33 (100%)	15 (100%)	
LP enhancement degree			1.000			1.000
Hypo-	71 (100%)	39 (100%)		33 (100%)	15 (100%)	

Data in parentheses are percentages except for special indications

*AP* arterial phase, *PVP* portal venous phase, *LP* late phases, *APHE* arterial phase hyperenhancement

* *p*_1_-value has a significant difference between SD and LD-type ICCs in the pilot cohort, or *p*_2_-value has a significant difference between SD and LD-type ICCs in the test cohort

^a^ Data are presented as the mean ± standard deviation

^b^ Data are presented as median (interquartile range)

### DCE-US quantitative analysis

**Fig. 4:**
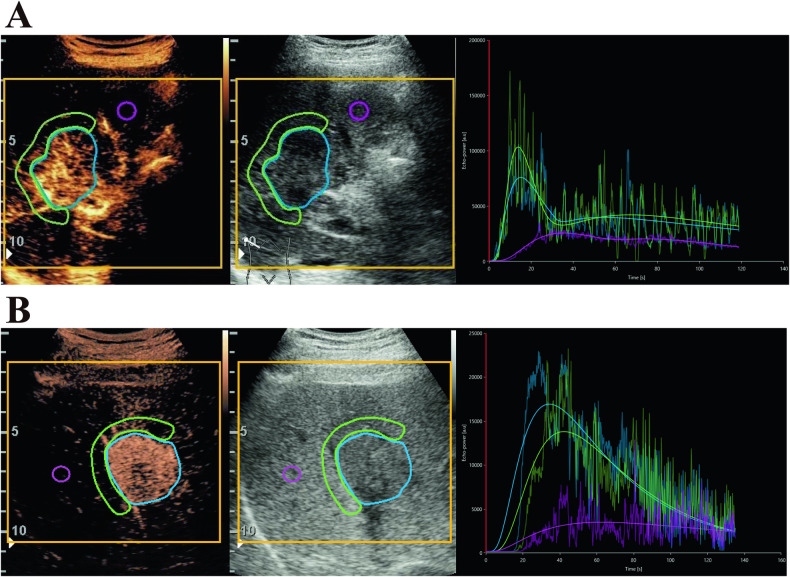
Dynamic contrast-enhanced ultrasound (DCE-US) perfusion analysis using the VueBox®. Blue regions of interest (ROIs) are placed around the whole intrahepatic cholangiocarcinoma (ICC) lesion, green ones at the margin of the ICC lesion, and pink ones in the surrounding liver parenchyma accordingly. Time-intensity curves (TICs) of the contrast agent in the ROIs are generated. **A** LD-type ICC, **B** SD-type ICC

**Fig. 5:**
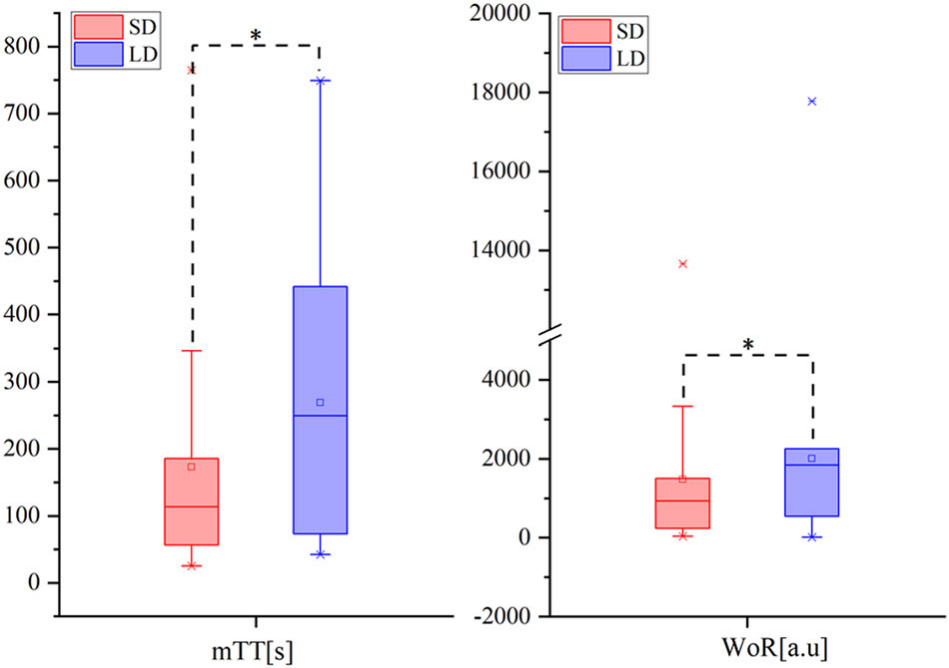
The mean transit time (mTT) and wash-out rate (WoR) of the margin of ICC lesions. Both mTT and WoR are significantly higher in the larger-duct (LD) group than in the small-duct (SD) group (*p < 0.05, *p* = 0.041, 0.007, respectively)

In the two cohorts, by univariate analysis, no difference was found in PE, RT, and AUC, of both the whole lesion and margin of lesion, between LD-type and SD-type ICCs (*p* > 0.05). Meanwhile, there was no significant difference in the variables of the surrounding liver parenchyma between the two subtypes (all *p* > 0.05). Further quantitative analysis demonstrated reliable results using VueBox® of all ICC lesions with QOF exceeding 75%, whose examples are shown in Fig. 4. In the pilot cohort, while comparing the two groups, mTT (289.92 ± 223.49 s vs. 172.94 ± 162.04 s, *p* = 0.041) and WoR (2003.96 ± 2977.73 a.u vs. 1476.30 ± 2404.42 a.u, *p* = 0.007) at margin of lesion was higher in LD-type group. Meanwhile, setting ROIs at the margin area of ICCs, FT (33.88 ± 19.80 s vs. 39.16 ± 16.92 s, *p* = 0.015), WoAUC (407,885.59 ± 282,999.15 a.u vs. 1,187,394.68 ± 2,327,139.41 a.u, *p* = 0.006) and WiWoAUC (596,801.30 ± 411,445.61 a.u vs. 1,643,893.92 ± 3,233,110.75 a.u, *p* = 0.014) were significantly lower in the LD-type group (Fig. 5). Similar findings were obtained in the test cohort, and the features which showed significant difference embraced mTT (285.71 ± 173.70 s vs. 177.43 ± 122.51 s, *p* < 0.001), WoR (2047.72 ± 2155.73 a.u vs. 1480.91 ± 5889.26 a.u, *p* < 0.001), FT (22.52 ± 18.20 s vs. 38.80 ± 23.09 s, *p* = 0.011), WoAUC (3,736,357.45 ± 6,788,198.20 a.u vs. 1,129,797.38 ± 2,605,483.40 a.u, *p* = 0.027) and WiWoAUC (5,506,335.10 ± 9,773,745.64 a.u vs. 1,678,171.83 ± 3,740,365.64 a.u, *p* = 0.014) at margin of lesion. No differences were found for the whole lesion in quantitative analysis (Table 3).

**Table 3:** Quantitative analysis of CEUS parameters for SD and LD

Variable	Pilot cohort			Test cohort		
	SD (*n* = 71)	LD (*n* = 39)	*p*_1_-value	SD (*n* = 33)	LD (*n* = 15)	*p*_2_-value
Mean contrast signal intensity, a.u						
Whole lesion	35,804.78 ± 79,900.86	36,510.62 ± 49,813.74	0.148	29,953.75 ± 33,107.08	46,012.34 ± 57,612.59	0.301
Margin of lesion	30,159.69 ± 64,878.16	22,592.74 ± 41,317.62	0.873	22,529.32 ± 30,680.70	28,088.86,847 ± 14,722.49	0.057
The surrounding liver parenchyma	20,701.55 ± 39,761.57	17,964.30 ± 25,223.02	0.344	12,578.60 ± 23,550.90	17,375.66 ± 39,664.96	0.920
Peak enhancement, a.u						
Whole lesion	62,020.50 ± 124,275.48	62,276.89 ± 102,313.83	0.637	83,274.84 ± 81,716.95	116,892.59 ± 143,350.63	0.344
Margin of lesion	41,052.39 ± 82,726.74	33,481.19 ± 54,938.91	0.646	64,493.88 ± 114,034.87	58,575.36 ± 27,892.74	0.073
The surrounding liver parenchyma	33,471.95 ± 66,511.85	34,975.26 ± 48,482.52	0.344	32,788.81 ± 51,653.58	51,159.40 ± 95,554.28	0.973
Wash-in area under the curve, a.u						
Whole lesion	540,350.84 ± 1,078,996.69	657,665.38 ± 1,043,283.71	0.429	652,194.04 ± 747,688.65	1,026,069.77 ± 1,575,107.18	0.443
Margin of lesion	541,359.57 ± 1,294,801.44	600,054.92 ± 1,368,930.62	0.888	625,212.27 ± 1,178,314.71	582,644.28 ± 272,942.51	0.066
The surrounding liver parenchyma	574,339.16 ± 1,092,114.39	472,885.25 ± 752,971.35	0.953	405,229.59 ± 1,060,321.61	489,439.74 ± 1,163,326.34	0.956
Rise time, s						
Whole lesion	14.62 ± 9.59	16.66 ± 8.50	0.075	13.58 ± 6.77	13.15 ± 7.52	0.798
Margin of lesion	18.35 ± 11.59	25.06 ± 17.77	0.139	17.99 ± 9.71	20.15 ± 7.39	0.261
The surrounding liver parenchyma	27.40 ± 17.10	23.54 ± 17.25	0.128	19.34 ± 15.53	17.95 ± 17.39	0.586
Mean transit time, s						
Whole lesion	153.02 ± 124.20	207.03 ± 165.70	0.188	119.42 ± 138.75	176.49 ± 210.57	0.868
Margin of lesion	172.94 ± 162.04	289.92 ± 223.49	0.041*	177.43 ± 122.51	285.71 ± 173.70	< 0.001*
The surrounding liver parenchyma	199.61 ± 191.03	159.22 ± 166.96	0.193	145.43 ± 166.78	158.00 ± 179.74	0.991
Time to peak, s						
Whole lesion	19.73 ± 12.53	20.96 ± 10.15	0.222	19.30 ± 8.98	18.71 ± 7.87	0.885
Margin of lesion	24.98 ± 14.81	31.27 ± 18.97	0.188	26.60 ± 12.09	26.73 ± 10.47	0.798
The surrounding liver parenchyma	37.51 ± 18.50	37.56 ± 21.08	0.748	35.44 ± 19.13	31.47 ± 23.20	0.133
Wash-in rate, a.u						
Whole lesion	9541.32 ± 17,710.69	9484.50 ± 17,280.76	0.602	9881.09 ± 14,318.91	9852.31 ± 5714.13	0.060
Margin of lesion	9192.64 ± 35,309.01	3828.92 ± 6074.33	0.360	9289.42 ± 19,341.27	9375.75 ± 10,227.80	0.261
The surrounding liver parenchyma	2945.70 ± 7091.02	4547.34 ± 8386.79	0.234	3521.47 ± 9462.32	4079.69 ± 9143.08	0.484
Wash-in perfusion index, a.u						
Whole lesion	41,473.40 ± 82,886.25	41,887.92 ± 67,664.22	0.611	54,279.61 ± 53,504.82	76,165.07 ± 92,547.35	0.380
Margin of lesion	27,470.66 ± 55,184.54	22,799.10 ± 38,904.53	0.660	27,407.12 ± 46,252.12	27,576.12 ± 23,700.60	0.151
The surrounding liver parenchyma	21,780.57 ± 41,576.91	22,488.16 ± 30,975.49	0.373	20,485.51 ± 32,217.77	33,092.29 ± 62,287.14	0.973
Wash-out area under the curve, a.u						
Whole lesion	1,374,000.90 ± 2,453,868.68	1,222,213.90 ± 1,663,661.37	0.584	1,686,540.59 ± 2,537,591.91	2,317,997.98 ± 3,328,974.13	0.368
Margin of lesion	1,187,394.68 ± 2,327,139.41	407,885.59 ± 282,999.15	0.006*	1,129,797.38 ± 2,605,483.40	3,736,357.45 ± 6,788,198.20	0.027*
The surrounding liver parenchyma	837,856.44 ± 1,435,598.45	811,306.94 ± 1,029,122.07	0.873	688,920.77 ± 1,763,849.60	1,323,979.79 ± 3,471,836.55	0.920
Wash-in and wash-out area under the curve, a.u						
Whole lesion	1,839,291.37 ± 3,251,180.72	1,687,035.92 ± 2,229,144.00	0.513	2,282,228.81 ± 3,090,582.19	3,361,831.15 ± 4,875,392.01	0.333
Margin of lesion	1,643,893.92 ± 3,233,110.75	596,801.30 ± 411,445.61	0.014*	1,678,171.83 ± 3,740,365.64	5,506,335.10 ± 9,773,745.64	0.014*
The surrounding liver parenchyma	1,228,703.30 ± 2,071,415.48	1,151,391.38 ± 1,317,792.28	0.761	1,093,159.28 ± 2,817,691.76	1,818,630.11 ± 4,633,087.86	0.956
Fall time, s						
Whole lesion	34.22 ± 19.04	34.73 ± 16.60	0.738	34.24 ± 23.83	28.63 ± 19.66	0.430
Margin of lesion	39.16 ± 16.92	33.88 ± 19.80	0.015*	38.80 ± 23.09	22.52 ± 18.20	0.011*
The surrounding liver parenchyma	47.08 ± 31.34	41.53 ± 27.91	0.285	34.09 ± 29.26	39.12 ± 29.92	0.483
Wash-out rate, a.u						
Whole lesion	2370.01 ± 3963.55	3181.24 ± 5033.43	0.144	2412.47 ± 3625.77	3037.90 ± 4016.72	0.193
Margin of lesion	1476.30 ± 2404.42	2003.96 ± 2977.73	0.007*	1480.91 ± 5889.26	2047.72 ± 2155.73	< 0.001*
The surrounding liver parenchyma	2028.52 ± 4187.17	2336.83 ± 5803.21	0.695	2023.78 ± 6066.39	2233.99 ± 5258.55	0.430

Data are presented as the mean ± standard deviation

* *p*_1_-value has a significant difference between SD and LD-type ICCs in the pilot cohort, or *p*_2_-value has a significant difference between SD and LD-type ICCs in the test cohort

According to the ROC analysis for the pilot cohort, the optimal cut-off value to predict LD-type ICC was estimated to be less than 788,507.73 a.u, 1,045,468.69 a.u, and 38.77 s for the WoAUC, WiWoAUC, and FT, and be more than 222.91 s and 1694.71 a.u for mTT and WoR of margin of lesions respectively (Supplement Table 2).

### Diagnostic performances of predictive model

Further multivariate analysis was conducted on the significant parameters of univariate analysis according to the pilot cohort, and the three parameters of intrahepatic bile duct dilation, mTT, and WoR were ultimately identified as independent factors for ICC subtype predictive models (Supplement Table 3). A regression predictive model which was established on this basis is as follows:

$$P=\frac{\exp ({\beta }_{0}+{\beta }_{1}{X}_{1}+{\beta }_{2}{X}_{2}+{\beta }_{3}{X}_{3})}{1+\exp ({\beta }_{0}+{\beta }_{1}{X}_{1}+{\beta }_{2}{X}_{2}+{\beta }_{3}{X}_{3})}$$

**Fig. 6:**
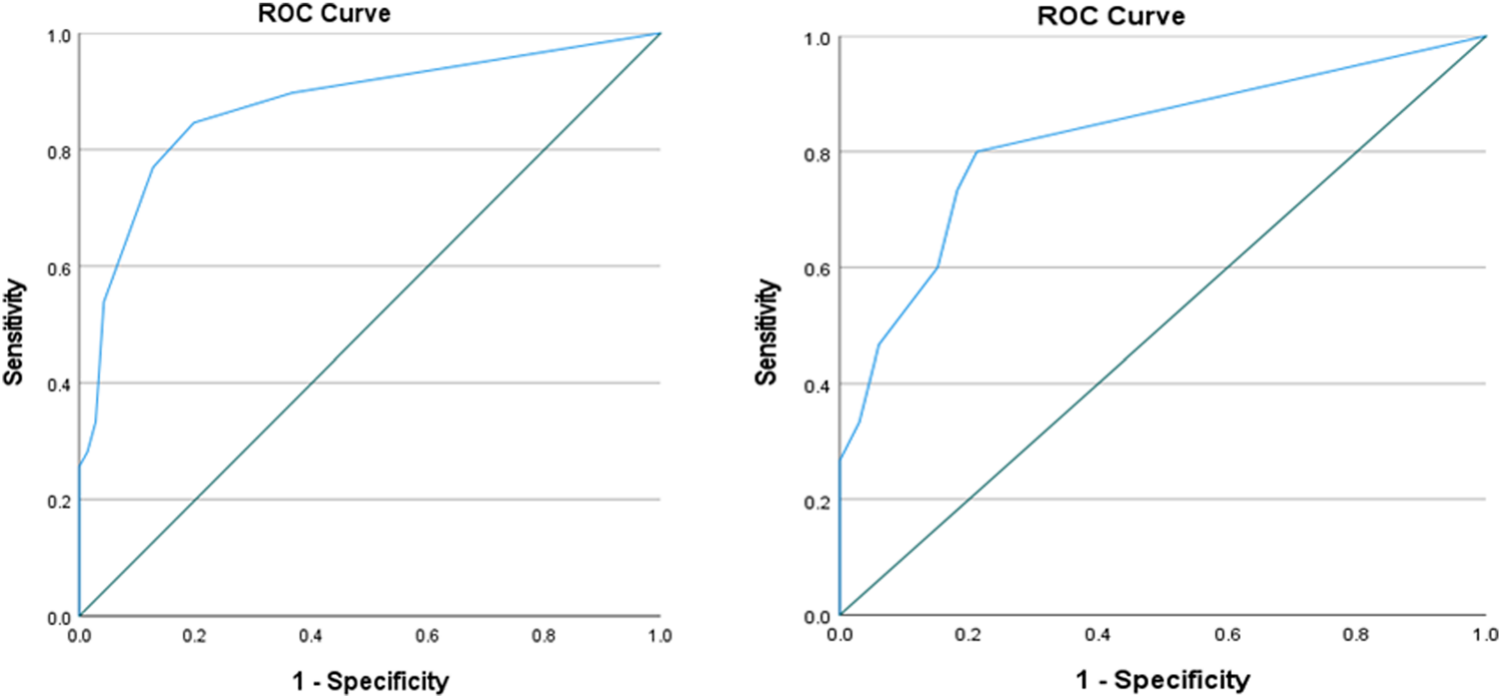
ROC curves for the performance of the predictive model in predicting ICC subtypes. The AUC value of the pilot cohort (left) is 0.875, with an accuracy of 0.818. Its sensitivity is 0.846, specificity is 0.803, PPV is 0.702, and NPV is 0.905. Meanwhile, the AUC value of the test cohort (right) is 0.833, with an accuracy of 0.792. Its sensitivity is 0.800, specificity is 0.788, PPV is 0.632, and NPV is 0.897

*X* refers to meaningful independent factors after binary logistic regression analysis, and each of these *β* values is shown in Table 4. The *p*-value of this formula represents the predicted probability value of LD-type ICC for each final inclusion lesion. When *p* was greater than 0.337, it was predicted as LD-type, and vice versa, it was SD-type. In this batch of data, the AUC value of the predictive model was 0.875, with an accuracy of 0.818. Its sensitivity was 0.846, specificity was 0.803, PPV was 0.702, and NPV was 0.905. Equally, using this formula to calculate *p*-value and diagnostic performances in the test cohort, the accuracy of the optimal prediction model was 0.792, with sensitivity, specificity, PPV, and NPV of 0.800, 0.788, 0.632, and 0.897, respectively. Table 5 and Fig. 6 provide the diagnostic performance of this logistic regression predictive model.

**Table 4:** Optimal predictive model

	*β*	SD	*p*-value	Odds ratio (95% CI)
The presence of intrahepatic bile duct dilation	2.675	0.621	< 0.001*	14.508 (4.291–49.046)
mTT, s	1.280	0.591	0.030*	3.596 (1.130–11.442)
WoR, a.u	2.365	0.627	< 0.001*	10.648 (3.115–36.392)
Constant	−2.549	0.471	< 0.001*	

*SD* standard deviation, *CI* confidence interval

* *p*-value has a significant difference between SD and LD-type ICCs (*p* < 0.05)

**Table 5:** Diagnostic performances of the predictive model

Predictive model	Pathology form			
	Pilot cohort		Test cohort	
	LD	SD	LD	SD
LD	33	14	26	3
SD	6	57	7	12
Sensitivity	0.846 (0.688, 0.936)		0.800 (0.514, 0.947)	
Specificity	0.803 (0.688, 0.884)		0.788 (0.606, 0.904)	
PPV	0.702 (0.549, 0.822)		0.632 (0.386, 0.828)	
NPV	0.905 (0.798, 0.961)		0.897 (0.715, 0.973)	
Accuracy	0.818 (0.733, 0.885)		0.792 (0.656, 0.885)	
AUC	0.875 (0.801, 0.950)		0.833 (0.695, 0.971)	

Data in parentheses are 95% confidence intervals

*SD* small-duct type of ICC, *LD* large-duct type of ICC, *PPV* positive predictive value, *NPV* negative predictive value, *AUC* area under the ROC curve

## Discussion

The study aimed to predict the pathologic subtype of ICC preoperatively through extracting and analyzing quantitative parameters based on DCE-US. There were significant differences in CEA, CA19-9, margin, intrahepatic bile duct dilation, mTT, WoAUC, WiWoAUC, FT, and WoR (*p* < 0.05) between LD-type and SD-type ICCs. Eventually, the presence of intrahepatic bile duct dilation, and higher values of mTT and WoR were identified as independent factors for predicting ICC subtypes through further binary logistic regression analysis. By incorporating these three factors into the regression predictive model and further validation in the prospective test cohort, the AUC of this model could achieve 0.833, which suggests that DCE-US has the potential to predict the two subtypes of ICCs and has clinical value.

ICC, as a primary liver malignancy, is divided into SD-type and LD-type based on different levels of origin. Due to the invasive procedure and limitations of biopsy, subclassification of ICCs by non-invasive imaging examinations would be of clinical value in preoperative differentiation. Park et al reported that MRI features reached a sensitivity of 59.6% and a specificity of 95.7% when combining two or more features. The US is one of the commonly used imaging methods for liver lesions in clinical practice, which is convenient and non-invasive. Due to the lack of clinical specificity in conventional gray-scale US features, characterization of ICC is often made through CEUS which can continuously and dynamically observe the blood supply of lesions. Meanwhile, DCE-US compensates for the subjectivity of CEUS by analyzing quantitative parameters to objectively evaluate the blood flow perfusion of the lesion. Therefore, we supposed that DCE-US may have the potential to predict the ICC subtype. In our study, clinical characters, conventional US and CEUS features, and the value of DCE-US quantitative analysis were estimated, and the differences in these variables between the two subtypes were analyzed.

The abnormally elevated CEA and CA 19-9 were more frequent in the LD-type ICC than in the SD-type. Fujita et al reported that hypovascular ICC showed a higher frequency of indistinct infiltrating pattern, perihilar-type ICC, and proximal bile duct dilatation, higher serum CA19-9 level, larger tumor size, more aggressive behavior, and more adverse outcomes, which were in accordance with the features of LD-type ICCs [13]. Zhang et al also reported that patients with double-negative AFP and CA19-9 had smaller tumor diameters and less aggressive performance, that is, an increase in CA19-9 is a predictor of more invasive tumor characteristics and worse clinical outcomes [25]. To sum up, elevated CEA and CA19-9 are more prone to be observed in LD-type ICCs whose prognosis is poorer than SD-type ICCs, which is consistent with our results [26, 27].

Pathologically, LD-type ICCs mostly originate from the second to third branches of hepatic bile ducts, and SD-type ICCs originate from the septal and interlobular bile ducts [28, 29]. Therefore, in gross classification, LD-type lesions often manifest in the perihilar location and SD-type lesions often in the peripheral liver parenchyma. However, due to the complex biliary anatomy in three dimensions, it is hard to discriminate subtypes of ICC just according to the location [10]. Similarly, based on our study results, the conclusion that the perihilar location of the lesion would more likely be LD-type ICCs cannot be drawn.

Our study revealed that significant differences can be found in margin through univariate analysis. A reasonable explanation is that LD-type ICC has stronger invasiveness, manifesting as a more blurred lesion margin on US. This is consistent with the previous studies that mentioned infiltrative contour on MRI and the higher proportion of positive resection margins in LD-type ICCs [6, 10, 30]. However, like parameters such as CA19-9, WoAUC, etc., ill-defined margin is not meaningful in multivariate analysis, indicating that these parameters are not independent factors for predicting ICC subtypes and therefore have not been included in the predictive model.

The presence of intrahepatic bile duct dilation was determined to be a significant US feature of LD-type ICC, which could be associated with the origin of LD-type from the intrahepatic large-duct group. Meanwhile, the bile duct dilation is also due to LD-type ICC being mostly characterized by biliary intraepithelial neoplasia and larger tumor size [1]. These results are in agreement with a previous study by Rhee et al, in which ductal-type MF-ICC, which most likely is LD-type, exhibited significantly more periductal tumor spread, presence of chronic biliary disease, and adjacent bile duct dilation [9].

In DCE-US, both LD-type and SD-type ICCs mostly exhibited hyperenhancement in AP, and all of them showed hypoenhancement in PVP and LP. Most of the lesions exhibited rim APHE, which is the characteristic feature of ICC, and displayed early wash-out onset within 60 s and marked wash-out degree within 2 min. Meanwhile, 14 CEUS quantitative parameters were selected, in which mTT, FT, WoAUC, WoR, and WiWoAUC had significant differences between the two subtypes. WoAUC and WiWoAUC reflect the total amount of contrast agent entering the lesion, and WoR reflects the clearance rate of contrast agent [31, 32]. The time-related parameter mTT is related to the residence time of the contrast agent microbubbles in ROIs and is also influenced by the injection method of the contrast agent [33]. For malignant tumors, infiltrating growth will destroy the surrounding vascular structure, and Aishima et al reported that perihilar-type ICC has few intratumoral arteries, resulting in less contrast agent entering the lesion [34, 35]. Meanwhile, because there are new tumor vessels in the lesion, which lack a basement membrane thereby loosening the connection between endothelial cells, the contrast agent diffuses rapidly from the vessels to the periparenchyma [36]. Therefore, compared with the SD-type, the WoAUC, and WiWoAUC of the LD-type are lower, FT is shorter and WoR is faster, which is consistent with the results in our study. However, there is no reasonable explanation for the longer mTT of LD-type, and our research results had discrepancies with the previous studies which showed no significant differences in mTT between advanced abdominal malignant tumors before and after the initiation of HIFU treatment, which required further validation [37].

There were some limitations of this study. First, the sample size in this study was relatively limited. However, the incidence of ICC is much lower than HCC and the number of 110 cases was statistically adequate to develop a predictive model in our study. Importantly, good prospective validation results were also obtained. Secondly, three different types of US equipment were used, making some US parameters inconsistent, which may cause confounding bias in the analysis process. A prospective multicenter study with unified US equipment in predicting the subtype of ICC should be carried out in the future.

In conclusion, DCE-US can be used to assess the subtypes of ICC lesions quantitatively, objectively, accurately, and preoperatively and the presence of intrahepatic bile duct dilation, mTT, and WoR are important parameters for the prediction of ICC subtype. Preoperative DCE-US-based imaging classification has the potential to supplement the subtype prediction of ICC.

### Supplementary information

Supplementary Information

## Data Availability

The datasets used or analyzed during the current study are available from the corresponding author upon reasonable request.

## Abbreviations

AFP: Alpha-fetoprotein
CA19-9: Carbohydrate antigen 19-9
CEA: Carcinoembryonic antigen
CEUS: Contrast-enhanced ultrasound
DCE-US: Dynamic contrast-enhanced ultrasound
FT: Fall time
HCC: Hepatocellular carcinoma
ICC: Intrahepatic cholangiocarcinoma
mTT: Mean transit time
PE: Peak enhancement
ROC: Receiver operating characteristic
RT: Rise time
TIC: Time intensity curve
WoAUC: Wash-out area under the curve
WoR: Wash-out rate

## References

[1] Banales JM, Marin JJG, Lamarca A et al (2020) Cholangiocarcinoma 2020: the next horizon in mechanisms and management. Nat Rev Gastroenterol Hepatol 17:557–588. 10.1038/s41575-020-0310-z 10.1038/s41575-020-0310-z PMC7447603 32606456

[2] Chun YS, Javle M (2017). Systemic and adjuvant therapies for intrahepatic cholangiocarcinoma. Cancer Control.

[3] Nagtegaal ID, Odze RD, Klimstra D (2020). The 2019 who classification of tumours of the digestive system. Histopathology.

[4] Aishima S, Fujita N, Mano Y (2011). Different roles of s100p overexpression in intrahepatic cholangiocarcinoma: carcinogenesis of perihilar type and aggressive behavior of peripheral type. Am J Surg Pathol.

[5] Nakanuma Y, Kakuda Y (2015). Pathologic classification of cholangiocarcinoma: new concepts. Best Pract Res Clin Gastroenterol.

[6] Aishima S, Oda Y (2015). Pathogenesis and classification of intrahepatic cholangiocarcinoma: different characters of perihilar large duct type versus peripheral small duct type. J Hepatobiliary Pancreat Sci.

[7] Group C S o L C C C. (2022) Chinese expert consensus on management of intrahepatic cholangiocarcinoma (2022 edn). Chin J Dig Surg. 10.3760/cma.j.cn115610-20220829-0047610.3760/cma

[8] Wee A (2011). Fine needle aspiration biopsy of hepatocellular carcinoma and hepatocellular nodular lesions: role, controversies and approach to diagnosis. Cytopathology.

[9] Rhee H, Kim MJ, Park YN, An C (2019). A proposal of imaging classification of intrahepatic mass-forming cholangiocarcinoma into ductal and parenchymal types: clinicopathologic significance. Eur Radiol.

[10] Park S, Lee Y, Kim H (2022). Subtype classification of intrahepatic cholangiocarcinoma using liver MR imaging features and its prognostic value. Liver Cancer.

[11] Xiao Y, Zhou C, Ni X (2023). Preoperative subcategorization based on magnetic resonance imaging in intrahepatic cholangiocarcinoma. Cancer Imaging.

[12] Nam JG, Lee JM, Joo I (2018). Intrahepatic mass-forming cholangiocarcinoma: relationship between computed tomography characteristics and histological subtypes. J Comput Assist Tomogr.

[13] Fujita N, Asayama Y, Nishie A (2017). Mass-forming intrahepatic cholangiocarcinoma: enhancement patterns in the arterial phase of dynamic hepatic ct-correlation with clinicopathological findings. Eur Radiol.

[14] Chen L, Xu H, Xie X (2010). Intrahepatic cholangiocarcinoma and hepatocellular carcinoma: differential diagnosis with contrast-enhanced ultrasound. Eur Radiol.

[15] Xu HX, Liu GJ, Lu MD (2006). Characterization of small focal liver lesions using real-time contrast-enhanced sonography: diagnostic performance analysis in 200 patients. J Ultrasound Med.

[16] Kang HJ, Kim JH, Joo I, Han JK (2020). Additional value of contrast-enhanced ultrasound (ceus) on arterial phase non-hyperenhancement observations (≥ 2 cm) of ct/mri for high-risk patients: focusing on the ct/mri li-rads categories lr-3 and lr-4. Abdom Radiol (NY).

[17] Wan P, Xue H, Liu C et al (2022) Transport-based anatomical-functional metric learning for liver tumor recognition using dual-view dynamic ceus imaging. IEEE Trans Biomed Eng. 10.1109/tbme.2022.3207473 10.1109/TBME.2022.3207473 36121950

[18] Parra Ramirez P, Santiago Hernando A, Barquiel Alcala B, Martin Rojas-Marcos P, Lisbona Catalan A, Alvarez Escola C (2019) Potential utility of contrast-enhanced ultrasound in the preoperative evaluation of primary hyperparathyroidism. J Ultrasound Med 38:2565–2571. 10.1002/jum.14949 10.1002/jum.14949 30693978

[19] Zhang Y, Zhou P, Tian SM, Zhao YF, Li JL, Li L (2017) Usefulness of combined use of contrast-enhanced ultrasound and ti-rads classification for the differentiation of benign from malignant lesions of thyroid nodules. Eur Radiol 27:1527–1536. 10.1007/s00330-016-4508-y 10.1007/s00330-016-4508-y PMC5334375 27525973

[20] Schwarze V, Marschner C, Negrao De Figueiredo G, Ingrisch M, Rubenthaler J, Clevert DA (2020) Single-center study: Dynamic contrast-enhanced ultrasound in the diagnostic assessment of carotid body tumors. Quant Imaging Med Surg 10:1739–1747. 10.21037/qims-19-920 10.21037/qims-19-920 PMC7417754 32879853

[21] Huang H, Zhu ZQ, Zhou ZG (2016). Contrast-enhanced transrectal ultrasound for prediction of prostate cancer aggressiveness: the role of normal peripheral zone time-intensity curves. Sci Rep.

[22] Goossen TE, de la Rosette JJ, Hulsbergen-van de Kaa CA, van Leenders GJ, Wijkstra H (2003). The value of dynamic contrast enhanced power doppler ultrasound imaging in the localization of prostate cancer. Eur Urol.

[23] Dong Y, Qiu Y, Yang D (2021). Potential application of dynamic contrast enhanced ultrasound in predicting microvascular invasion of hepatocellular carcinoma. Clin Hemorheol Microcirc.

[24] Cho MK, Moon SH, Song TJ (2018). Contrast-enhanced endoscopic ultrasound for differentially diagnosing autoimmune pancreatitis and pancreatic cancer. Gut Liver.

[25] Zhang X, Zhou Y, Wu Z (2021). Double-negative α-fetoprotein and carbohydrate antigen 19-9 predict a good prognosis in intrahepatic cholangiocarcinoma: a propensity score matching analysis. Clin Transl Gastroenterol.

[26] Ma B, Meng H, Shen A (2021). Prognostic value of inflammatory and tumour markers in small-duct subtype intrahepatic cholangiocarcinoma after curative-intent resection. Gastroenterol Res Pract.

[27] Hayashi A, Misumi K, Shibahara J (2016). Distinct clinicopathologic and genetic features of 2 histologic subtypes of intrahepatic cholangiocarcinoma. Am J Surg Pathol.

[28] Banales JM, Cardinale V, Carpino G (2016). Expert consensus document: cholangiocarcinoma: current knowledge and future perspectives consensus statement from the European Network for the Study of Cholangiocarcinoma (ENS-CCA). Nat Rev Gastroenterol Hepatol.

[29] Akita M, Sofue K, Fujikura K (2019). Histological and molecular characterization of intrahepatic bile duct cancers suggests an expanded definition of perihilar cholangiocarcinoma. HPB (Oxford).

[30] Nishihara Y, Aishima S, Hayashi A (2009). Cd10+ fibroblasts are more involved in the progression of hilar/extrahepatic cholangiocarcinoma than of peripheral intrahepatic cholangiocarcinoma. Histopathology.

[31] Frohlich E, Muller R, Cui XW, Schreiber-Dietrich D, Dietrich CF (2015). Dynamic contrast-enhanced ultrasound for quantification of tissue perfusion. J Ultrasound Med.

[32] Nylund K, Saevik F, Leh S, Pfeffer F, Hausken T, Gilja OH (2019). Interobserver analysis of ceus-derived perfusion in fibrotic and inflammatory crohn’s disease. Ultraschall Med.

[33] Wiesinger I, Beyer LP, Zausig N (2018). Evaluation of integrated color-coded perfusion analysis for contrast-enhanced ultrasound (CEUS) after percutaneous interventions for malignant liver lesions: first results. Clin Hemorheol Microcirc.

[34] Aishima S, Iguchi T, Nishihara Y (2009). Decreased intratumoral arteries reflect portal tract destruction and aggressive characteristics in intrahepatic cholangiocarcinoma. Histopathology.

[35] Wendl CM, Janke M, Jung W, Stroszczysnski C, Jung EM (2015). Contrast-enhanced ultrasound with perfusion analysis for the identification of malignant and benign tumours of the thyroid gland. Clin Hemorheol Microcirc.

[36] Fang J, Islam W, Maeda H (2020). Exploiting the dynamics of the EPR effect and strategies to improve the therapeutic effects of nanomedicines by using EPR effect enhancers. Adv Drug Deliv Rev.

[37] Zuo D, Liu LX, Zhang Q (2022). Clinical value of dynamic contrasted enhanced ultrasound in monitoring therapeutic effect of high-intensity focused ultrasound in abdominal malignant tumor. Fudan Univ J Med Sci.

