# Supplementary material for: Subtype prediction of intrahepatic cholangiocarcinoma using dynamic contrast-enhanced ultrasound
Source: Insights Imaging. 2024 May 16;15:119. doi: 10.1186/s13244-024-01683-y (PMC11098973; doi:10.1186/s13244-024-01683-y)
Supplement: Supplementary file 1 — Supplementary Information [file 13244_2024_1683_MOESM1_ESM.pdf]

Subtype Prediction of Intrahepatic Cholangiocarcinoma Using Dynamic Contrast-enhanced Ultrasound

ELECTRONIC SUPPLEMENTARY MATERIAL

Supplement Table 1. Comparison of quantitative parameters in different instruments.

Parameters	Subtypes and Instruments							
	SD (n=71)				LD (n=39)			
	Samsung RS80A (n=39)	Mindray Resona 7s (n=15)	EPIQ7 (n=17)	*p* value	Samsung RS80A (n=25)	Mindray Resona 7s (n=8)	EPIQ7 (n=6)	*p* value
Mean contrast signal intensity of the whole lesion, a.u	12407.94 ±9513.89	41861.92 ±58225.38	67527.95 ±119195.80	0.019	15709.59 ±13412.33	61645.10 ±49384.75	85665.53 ±84843.33	0.002
Mean contrast signal intensity at the margin of lesion, a.u	10591.13 ±9456.77	33201.14 ±46301.15	56981.42 ±96166.90	0.009	12357.79 ±12485.83	25160.41 ±25641.48	56578.46 ±87972.44	0.118
Mean contrast signal intensity in the surrounding liver parenchyma, a.u	7728.60 ±8107.35	21809.71 ±18601.40	38612.70 ±58240.19	0.027	9450.56 ±7733.07	40779.14 ±46977.69	25555.69 ±24630.17	0.123
Peak enhancement of the whole lesion, a.u	23707.39 ±19141.28	111531.11 ±91215.11	108312.25 ±184106.38	0.011	22445.84 ±19174.96	90613.07 ±65923.34	176194.44 ±194407.25	0.001
Peak enhancement at the margin of lesion, a.u	17114.13 ±15288.64	77148.66 ±63904.65	69238.37 ±122945.54	0.001	16738.94 ±16300.58	40543.88 ±38139.98	86212.23 ±110128.08	0.042
Peak enhancement in the surrounding liver parenchyma, a.u	13390.70 ±13842.61	36509.71 ±33166.63	61008.31 ±98524.55	0.001	17703.63 ±13842.53	53544.47 ±59365.92	78090.41 ±82353.50	0.042
Wash-in area under the curve of the whole lesion, a.u	217767.22 ±177700.82	1122265.56 ±1446141.24	906533.06 ±1549851.83	0.013	267261.59 ±268691.73	1060667.09 ±1388825.18	1648962.94 ±1644643.29	0.011
Wash-in area under the curve at the margin of lesion, a.u	210893.88 ±196054.53	946767.54 ±1343406.66	943735.64 ±1934396.35	0.020	354192.77 ±468786.54	234902.82 ±247897.81	1843286.10 ±2952512.94	0.240
Wash-in area under the curve in the surrounding liver parenchyma, a.u	240059.22 ±265398.49	399823.48 ±244514.90	1064874.16 ±1603430.73	0.038	338709.00 ±399076.83	671357.15 ±1227056.78	753614.23 ±1111374.22	0.814
Rise time of the whole lesion, s	14.86±11.17	13.13±8.26	14.50±7.41	0.759	17.63±8.32	14.57±7.31	15.25±10.79	0.333
Rise time at the margin of lesion, s	18.48±12.23	14.50±9.30	18.72±11.22	0.697	29.91±18.28	9.18±1.63	23.62±16.26	0.004
Rise time in the surrounding liver	30.23±19.19	23.18±11.76	24.06±14.08	0.490	28.21±16.91	14.46±8.17	15.98±20.48	0.017

parenchyma, s								
Mean transit time of the whole lesion, s	147.71±118.54	75.22±42.40	171.53±136.83	0.232	238.25±165.23	141.85±153.89	160.70±171.70	0.164
Mean transit time at the margin of lesion, s	168.78±148.51	116.69±63.16	186.77±189.46	0.969	344.10±179.49	42.61±27.54	343.75±315.83	0.003
Mean transit time in the surrounding liver parenchyma, s	226.78±217.18	119.96±79.24	173.13±157.45	0.762	198.17±172.52	69.90±51.74	109.44±190.51	0.017
Time to peak of the whole lesion, s	19.46±13.20	17.93±11.21	20.37±12.10	0.907	21.48±10.43	18.72±8.18	21.38±11.93	0.835
Time to peak at the margin of lesion, s	24.90±15.53	30.75±20.03	24.27±13.38	0.868	36.14±20.20	14.91±4.06	30.21±14.75	0.006
Time to peak in the surrounding liver parenchyma, s	39.98±20.12	32.74±17.74	34.74±16.19	0.426	44.06±21.87	23.26±10.15	28.62±17.04	0.011
Wash-in rate of the whole lesion, a.u	4023.32 ±4143.15	16524.59 ±19183.47	16229.49 ±25506.31	0.047	4163.55 ±5795.36	12225.48 ±9663.96	25746.94 ±35046.73	0.003
Wash-in rate at the margin of lesion, a.u	2616.05 ±3496.24	8326.24 ±5651.41	18476.66 ±55313.02	0.003	1786.45 ±2084.25	6752.19 ±7040.96	8200.16 ±10820.74	0.012
Wash-in rate in the surrounding liver parenchyma, a.u	1181.96 ±1481.30	3952.81 ±5269.99	5258.46 ±10676.97	0.001	1509.37 ±1549.40	6786.27 ±5772.28	13158.32 ±16484.63	0.004
Wash-in perfusion index of the whole lesion, a.u	15630.27 ±12404.72	72961.49 ±59894.60	72970.88 ±122702.33	0.011	15586.54 ±13497.41	61309.20 ±45575.52	116400.17 ±128027.98	0.001
Wash-in perfusion index at the margin of lesion, a.u	11337.71 ±10050.69	49133.38 ±41014.07	46846.88 ±82045.22	0.001	11674.05 ±11711.68	25723.74 ±24177.68	59606.77 ±79664.30	0.059
Wash-in perfusion index in the surrounding liver parenchyma, a.u	8833.41 ±9078.71	23736.79 ±21746.68	39534.64 ±61184.40	0.002	11454.63 ±8996.40	35550.35 ±41891.41	48831.43 ±49821.16	0.044
Wash-out area under the curve of the whole lesion, a.u	673242.88 ±548645.84	4269578.51 ±6568072.16	1936402.78 ±2848655.63	0.011	812952.39 ±781296.59	1259535.86 ±841728.94	2646540.21 ±3363429.15	0.100
Wash-out area under the curve at the margin of lesion, a.u	672521.36 ±580525.10	2344115.34 ±3537007.41	1739293.84 ±3358425.19	0.115	364014.78 ±192173.50	489755.88 ±529623.45	482696.77 ±237980.98	0.597
Wash-out area under the curve in the surrounding liver parenchyma, a.u	725809.87 ±827672.76	1055971.88 ±483484.51	1351909.52 ±2114863.94	0.095	592126.70 ±998102.81	345877.76 ±201396.85	502932.50 ±370622.89	0.720
Wash-in and wash-out area under the curve of	913967.68 ±738401.46	5391843.97 ±8011020.49	2620627.56 ±3970703.42	0.009	1088442.34 ±946395.51	1753070.41 ±1088164.92	3758835.66 ±4494250.57	0.083

the whole lesion, a.u								
Wash-in and wash-out area under the curve at the margin of lesion, a.u	934700.36 ±802944.40	3290882.95 ±4879571.54	2396415.09 ±4672451.70	0.121	527724.32 ±269175.27	724658.72 ±776831.44	715647.37 ±351330.86	0.570
Wash-in and wash-out area under the curve in the surrounding liver parenchyma, a.u	1013999.07 ±1074027.82	1455795.36 ±724738.48	1995014.68 ±3057457.23	0.135	846305.74 ±1274221.11	533861.30 ±310602.77	859709.86 ±681641.44	0.604
Fall time of the whole lesion, s	36.67±23.56	30.80±19.32	31.29±9.90	0.808	38.41±18.78	29.71±9.78	26.61±8.71	0.313
Fall time at the margin of lesion, s	42.29±18.86	30.81±23.20	35.99±12.13	0.421	37.91±21.26	18.41±4.52	34.96±17.51	0.004
Fall time in the surrounding liver parenchyma, s	51.72±30.73	56.10±31.15	41.77±21.81	0.280	43.83±34.09	22.98±11.00	16.72±13.17	0.003
Wash-out rate of the whole lesion, a.u	1319.18 ±1337.66	4514.94 ±4615.20	3527.24 ±5672.61	0.002	1630.60 ±1278.78	3704.14 ±3404.77	8196.33 ±10131.30	0.010
Wash-out rate at the margin of lesion, a.u	715.81 ±582.51	3588.89 ±2329.55	2233.75 ±3437.49	0.003	1355.86 ±894.25	2750.07 ±2832.06	3572.50 ±6298.21	0.774
Wash-out rate in the surrounding liver parenchyma, a.u	818.38 ±788.62	1159.60 ±1475.60	2802.25 ±7377.94	0.045	1466.51 ±1426.72	3063.40 ±2524.19	10271.80 ±12079.43	0.003

Note. — Data are presented in mean ± standard deviation.

Supplement Table 2. Converting quantitative into binary variables.

Parameters	AUROC	Youden index	Cutoff value	Value tending to LD
Margin				
mTT, s	0.618 (0.494, 0.742)	0.376	222.91	> cutoff value
WoAUC, a.u	0.659 (0.557, 0.761)	0.456	788507.73	< cutoff value
WiWoAUC, a.u	0.642 (0.539, 0.746)	0.433	1045468.69	< cutoff value
FT, s	0.641 (0.532, 0.750)	0.370	38.77	< cutoff value
WoR, a.u	0.655 (0.538, 0.772)	0.466	1694.71	> cutoff value

Note. —Data in parentheses are 95% confidence intervals.

mTT, mean transit time; AUROC, area under the ROC curve; WoAUC, wash-out area under the curve; WiWoAUC, wash-in and wash-out area under the curve; FT, fall time; WoR, wash-out rate.

Supplement Table 3. Univariate and multivariate analyses of parameters in predicting ICC subtypes.

Parameters	Univariate			Multivariate		
	OR	95% CI	*p* value	OR	95% CI	*p* value
Age, years	1.032	0.990, 1.075	0.133			
Male/female	1.937	0.875, 4.287	0.103			
Nodule size (IQR), mm	0.994	0.979, 1.011	0.492			
History of hepatitis	0.603	0.259, 1.404	0.241			
AFP > 20, ng/mL	0.784	0.191, 3.222	0.735			
CEA > 5, ng/mL	5.120	1.849, 14.174	0.002*			
CA19-9 > 34, U/mL	3.259	1.435, 7.402	0.005*			
Location	0.465	0.199, 1.091	0.079			
Echo intensity	1.412	0.929, 2.145	0.106			
Margin	10.179	1.290, 80.320	0.028*			
Hepatic background	0.873	0.549, 1.387	0.564			
The presence of intrahepatic bile duct dilation	10.191	3.862, 26.895	< 0.001*	14.508	4.291, 49.046	< 0.001*
AP Enhancement pattern	1.091	0.546, 2.179	0.805			
Enhancement onset time ,s	1.038	0.952, 1.132	0.394			
AP peak time, s	1.044	0.971, 1.123	0.242			
Wash-out onset time (IQR), s	1.011	0.980, 1.042	0.502			
Wash-out degree	0.553	0.265, 1.152	0.113			
Mean contrast signal intensity of the whole lesion, a.u	2.065	0.930, 4.583	0.075			
Mean contrast signal intensity at the margin of lesion, a.u	1.758	0.757, 4.083	0.190			
Peak enhancement of the whole lesion, a.u	1.384	0.619, 3.095	0.429			
Peak enhancement at the margin of lesion, a.u	3.543	1.380, 9.095	0.009*			
Wash-in area under the curve of the whole lesion, a.u	1.983	0.800, 4.915	0.139			
Wash-in area under the curve at the margin of lesion, a.u	2.052	0.786, 5.361	0.142			
Rise time of the whole lesion, s	2.882	1.284, 6.469	0.010*			
Rise time at the margin of lesion, s	1.599	0.644, 3.974	0.312			
Mean transit time of the whole lesion, s	2.600	1.147, 5.896	0.022*			
Mean transit time at the margin of lesion, s	5.269	2.226, 12.471	< 0.001*	3.596	1.130, 11.442	0.030*
Time to peak of the whole lesion, s	2.879	0.324, 25.564	0.343			
Time to peak at the margin of lesion, s	1.021	0.997, 1.046	0.092			
Wash-in rate of the whole lesion, a.u	1.857	0.738, 4.671	0.189			
Wash-in rate at the margin of lesion, a.u	2.378	0.954, 5.925	0.063			

Wash-in perfusion index of the whole lesion, a.u	1.383	0.591, 3.239	0.455			
Wash-in perfusion index at the margin of lesion, a.u	2.835	1.143, 7.035	0.025*			
Wash-out area under the curve of the whole lesion, a.u	1.550	0.687, 3.500	0.292			
Wash-out area under the curve at the margin of lesion, a.u	19.029	4.259, 85.025	< 0.001*			
Wash-in and wash-out area under the curve of the whole lesion, a.u	1.465	0.656, 3.274	0.352			
Wash-in and wash-out area under the curve at the margin of lesion, a.u	10.076	3.239, 31.339	< 0.001*			
Fall time of the whole lesion, s	1.679	0.755, 3.731	0.204			
Fall time at the margin of lesion, s	5.571	2.172, 14.291	< 0.001*			
Wash-out rate of the whole lesion, a.u	5.742	2.338, 14.103	< 0.001*			
Wash-out rate at the margin of lesion, a.u	11.832	4.332, 32.313	< 0.001*	10.648	3.115, 36.392	< 0.001*

Note. —**p*-value has significant difference between SD and LD-type ICCs.

Variables with $p < 0.05$ on univariate logistic regression analysis were utilized as input variables for multivariate logistic regression analysis.

SD, small-duct type of ICC; LD, large-duct type of ICC; AFP, alpha-fetoprotein; CEA, carcinoembryonic antigen; CA19-9, carbohydrate antigen 19-9; AP, arterial phase; PVP, portal venous phase; LP, late phases; APHE, arterial phase hyperenhancement; OR, odds ratio.
